# Incidence of High Risk and Malignant Pathological Findings in Transgender and Gender Diverse Individuals Undergoing Gender-Affirming Mastectomy

**DOI:** 10.1245/s10434-024-16005-1

**Published:** 2024-08-07

**Authors:** Rachel L. McCaffrey, Andrew J. James, Ricardo A. Torres-Guzman, Jamin K. Addae, Lauren E. Sullivan, Elianna Dash, Hanna Slutsky, Zoe R. Finer, Patrick E. Assi, Salam Al-Kassis

**Affiliations:** 1 Division of Surgical Oncology and Endocrine Surgery, Department of Surgery, Vanderbilt University Medical Center, Nashville, TN USA; 2 Department of Plastic Surgery, Vanderbilt University Medical Center, Nashville, TN USA; 3 Oncologic Surgery, OSF Healthcare, Peoria, IL USA; 4 Vanderbilt University School of Medicine, Nashville, TN USA

**Keywords:** Top surgery, Breast cancer screening, Gender affirming hormone therapy, Incidental breast pathology, Top surgery

## Abstract

**Background:**

Concrete, data-driven guidelines for breast cancer screening among the transgender and gender diverse (TGD) population is lacking. The present study evaluates possible associations of gender-affirming hormone therapy (GAHT) on incidental breast pathology findings in trans-masculine patients to inform decision making about breast cancer screening.

**Patients and Methods:**

This was a retrospective cohort study of patients who had gender-affirming mastectomy or breast reduction at a single center from July 2019 to February 2024. A total of 865 patients met the inclusion criteria. Gender-affirming testosterone therapy and length of exposure were evaluated to seek differences in post-operative pathology findings.

**Results:**

The median age at the time of surgery was 27 years [interquartile range (IQR) 21–30]. Most participants identified as female to male (658, 75.6%). A significant portion of the participants (688, 79.2%) were undergoing testosterone therapy at the time of surgery, with the median duration of testosterone use prior to surgery being 14 months (IQR 4–29). High risk or malignant findings were noted in pathology results for 12 of 1730 breasts (0.7%). Ordered logistic regression found that duration of testosterone therapy was not associated with increasing severity of incidental breast pathology. Additionally, patients under 25 years of age were 70% less likely to have any incidental finding on pathological evaluation than older patients [odds ratio (OR) 0.3, *p* < 0.01, confidence interval (CI) 0.18–0.50].

**Conclusions:**

The present study found that patients undergoing GAHT should not be screened for breast cancer with increased frequency compared with cis-gender women. Additionally, it may be appropriate for trans women under the age of 25 with normal breast cancer risk to forego pathological breast tissue examination.

Gender affirming surgeries in the transgender and gender diverse (TGD) population are on the rise. With approximately 1.6% of the adult population and up to 5% of young adult population in the USA identifying as TGD, the number of gender affirming surgeries will likely continue to increase.^1,2^ In 2020, the American Society of Plastic Surgeons reported that 8548 top surgeries were performed on TGD individuals with a 2024 investigation finding that roughly 24,000 patients had gender-affirming top surgery between 2016 and 2019.^3,4^

Gender-affirming mastectomies (GAM) at our institution aim to tailor the chest to affirm the patient’s gender identity while preserving local vasculature and lymphatic systems without necessitating total breast tissue removal in all patients. Other groups perform GAM cases in tandem with oncologic breast surgeons to remove all breast tissue as an oncologic mastectomy in high risk or known malignancy cases. These surgeries, coupled with testosterone as gender affirming homrone therapy (GAHT), introduce complex variables affecting breast cancer risk. GAHT, associated with lobular atrophy, theoretically benefits long-term breast cancer risk reduction. However, transmasculine patients on GAHT are found to have a higher breast cancer incidence than cisgender men, yet lower than cisgender women.^5,6^ Concurrently, breast reduction surgeries are linked to a decreased breast cancer risk, presumably due to the removal of hormone-responsive tissue.^7^ This intricate interplay between surgical techniques, hormonal treatments, and inherent risk factors contributes to the absence of standardized breast cancer screening guidelines for TGD patients with some studies identifying that less than 50% of these patients undergo age-appropriate breast cancer screening, which leaves a marginalized group without equitable cancer surveillance.^8^

Current surgical and radiological practices lack consensus on preoperative breast cancer screening and pathological analysis for gender-affirming surgeries. The current guidelines, primarily based on a single study and expert opinion, offer minimal direction, particularly for patients undergoing GAM, for whom screening is deemed “usually not appropriate” beyond certain age thresholds.^9,10^ In addition to ambiguous screening guidelines, the necessity and efficacy of routine pathological examinations for transgender individuals remain underexplored likely due to low patholgic incidence, which would require further surgical intervention, and the cost to patients undergoing GAM, which is often an out-of-pocket due to poor insurance coverage.^11^

This procedural ambiguity not only highlights the need for targeted research aimed at developing informed screening protocols and pathologic analysis protocols, but also underscores our study’s rationale. We hypothesize that through a detailed examination of gender-affirming top surgery specimens, significant insights into the nuanced breast cancer risks faced by TGD individuals can be garnered, paving the way for more inclusive, evidence-based clinical guidelines for tissue pathologic analysis and breast cancer screening.

## Patients and Methods

### Participants, Study Design, and Current Protocols

This retrospective study was conducted at Vanderbilt University Medical Center (VUMC) from July 2019 to February 2024, under institutional review board (IRB) approval number 231834. A total of 1340 patient records who received any type of transgender surgery care were initially screened, from which 865 underwent GAM and were selected for data extraction. Our inclusion criteria identified participants who underwent GAM with a primary diagnosis of gender dysphoria. Records were excluded if medical documentation was incomplete, tissue pathologic analysis was not performed, or if participants declined release of their medical data for research purposes at any time. Currently, all patients who undergo GAM at our institution have tissue pathology performed and no patients who underwent GAM during the study period had incomplete pathology results. The data for the study were securely managed and stored using the RedCap system, ensuring confidentiality.

Currently, all gender-affirming mastectomy candidates must meet the World Professional Association for Transgender Health criteria to be offered a procedure. Additionally, breast cancer risk is assessed using the Breast Cancer Risk Assessment Tool (BCRAT) from the National Cancer Institute. Patients of concern are offered additional mammographic or magnetic resonance imaging (MRI) screening prior to GAM.

### Data Collection

The data collection included patient demographics such as age, race, gender identity, medical and family histories, history of substance use, history of testosterone therapy, and genetic testing. Hormone use was captured directly from prescribing provider’s orders when available, or from review of patient encounter notes as ascertaining hormone therapy history is a standard part of the intake for patients establishing care with our gender-affirming providers. Specific breast pathology was collected and included benign lesions, high risk atypical lesions, and cancer data including disease staging and hormone receptor status, as well as surgical and adjuvant treatment details. Follow-up information aimed at capturing long-term outcomes, including survival status and methods of local surveillance, was also included. This provided a comprehensive overview of the breast cancer care continuum for transgender and gender-diverse individuals.

### Statistical Analysis

We performed statistical analyses using commercially available software (STATA statistical software version 18.0, StataCorp). A significance level of *p* < 0.05 was adopted for all statistical tests. An ordered logistic regression was performed to determine if age, gender, smoking status, alcohol use, body mass index (BMI), personal history of cancer, family history of cancer, or testosterone therapy were associated with increased severity of detected lesions. Patients with completely benign pathology received a cancer severity score of 0, those with nonproliferative lesions received a score of 1, those with proliferative disease without atypia a score of 2, proliferative disease with atypia received a score of 3, and those with malignancies were scored as a 4.

## Results

Our study population was comprised of 865 individuals (1610 breasts) with a median age at the time of surgery of 27 years [interquartile range (IQR) = 21–30]. Most participants identified as female to male (658, 75.6%), followed by nonbinary (181, 20.8%), with a small number identifying as female (2, 0.2%) or other (28, 3.2%). The ethnic composition was predominantly Caucasian (699, 80.1%), with smaller representations from African American (76, 8.8%), Latin-x (42, 4.8%), Asian (18, 2.1%), Native American/Alaskan Native (9, 1.0%), or other (21, 2.4%) backgrounds.

Concerning body mass index (BMI) at the time of surgery, the distribution was relatively spread across different ranges, with 25.5% of patients having a BMI of 26–30 kg/m^2^, followed closely by 25.2% in the 21–25 kg/m^2^ range, and 18.5% in the 31–35 kg/m^2^ range. Smaller percentages were distributed among the other BMI categories, reflecting a broad diversity in body compositions within our study cohort.

A significant portion of the participants (688, 79.2%) were undergoing testosterone therapy at the time of surgery, with the median duration of testosterone use prior to surgery being 14 months with an inner quartile range of 4–29 months. The rate of oophorectomy in this population was low, at 1.7%, which aligns with the selection criteria focusing primarily on top surgery. Other forms of hormone suppression or replacement were uncommon, indicating that testosterone therapy was the predominant hormone treatment in this group (Table 1).

**Table 1:** Demographics of gender affirming mastectomy patients from July 2019 to February 2024

Feature	Category	Number of patients
*N* = 865 patients		
Age at surgery, median (IQR)	25 (21, 30)	
Gender	Male	163 (18.8%)
	Female	2 (0.2%)
	Nonbinary	181 (20.9%)
	Other	28 (3.2%)
	Female to male	491 (56.8%)
Race	White	699 (80.1%)
	Black	76 (8.8%)
	Hispanic or Latino	42 (4.9%)
	Asian	18 (2.1%)
	Native American/Alaskan Native	9 (1.0%)
	Other	21 (2.4%)
Tobacco use	Nonsmoker	555 (64.2%)
	Current smoker	107 (12.4%)
	Former smoker	201 (23.2%)
	Not specified	2 (0.2%)
Alcohol use	No alcohol use	424 (49.0%)
	social alcohol use (1–2 times per month)	143 (16.5%)
	1–7 drinks per week	238 (27.5%)
	> 7 drinks per week	17 (2.0%)
	Not specified	43 (5.0%)
BMI at time of surgery	Not specified	22 (2.5%)
	15–20	56 (6.5%)
	21–25	218 (25.2%)
	26–30	221 (25.5%)
	31–35	161 (18.6%)
	36–40	103 (11.9%)
	41–45	54 (6.2%)
	46–50	22 (2.5%)
	> 50	8 (0.9%)
History of testosterone therapy	No	176 (20.3%)
	Yes	689 (79.7%)
Duration of testosterone therapy (in months), median (IQR)	14 (4, 29)	
History of oophorectomy		15 (1.7%)

In the analysis of pathologic findings, we observed a spectrum of lesions with varying implications for breast cancer risk. Among the specimens evaluated, benign nonproliferative lesions constituted 6.2% of the findings, encompassing fibrocystic changes (2.7%), fibroadenoma (1.5%), and apocrine metaplasia (2%). Proliferative diseases without atypia, indicative of a slightly elevated risk for developing breast cancer, were identified in 2.4% of cases, including usual ductal hyperplasia (1.4%), sclerosing adenosis (0.2%), columnar cell changes (0.4%), and intraductal papilloma (0.4%). Moreover, the presence of proliferative disease with atypia, which confers a higher risk for breast cancer, was noted in a smaller fraction of the population, with atypical ductal hyperplasia and atypical lobular hyperplasia being found in 0.1% and 0.2% of specimens, respectively. Malignant lesions were rare, with only two cases identified: one instance of ductal carcinoma in situ (DCIS) and one case of Paget’s disease, each constituting 0.1% (see Table 2). All malignant lesions and 50% of proliferative lesions with atypia were found in patients aged 35 years or older (see Table 3).

**Table 2:** Pathologic findings collected from electronic medical records of gender-affirming mastectomy patients

Pathology	*N* (%)
Benign breast parenchyma	1610 (93.06)
Benign nonproliferative	83 (4.80)
Fibrocystic changes	46 (2.70)
Fibroadenoma	26 (1.5)
Apocrine metaplasia	35 (2.02)
Proliferative disease without atypia	31 (1.79)
Usual ductal hyperplasia	24 (1.39)
Sclerosing adenosis	3 (0.17)
Columnar cell change	6 (0.35)
Papilloma	7 (0.40)
Proliferative disease with atypia	4 (0.23)
Atypical ductal hyperplasia	1 (0.06)
Atypical lobular hyperplasia	3 (0.17)
Malignant lesions	2 (0.12)
Ductal carcinoma in situ (DCIS)	1 (0.06)
Paget’s disease	1 (0.06)

**Table 3:** Pathology results of each breast sample by age category

Age			Pathology results		
	Benign breast parenchyma	Benign nonproliferative	Proliferative disease without atypia	Proliferative disease with atypia	Malignant lesions
< 25	734 (95%)	30 (4%)	6 (0.8%)	2 (0.3%)	0 (0%)
25–34	674 (88%)	64 (9%)	26 (3%)	2 (0.3%)	0 (0%)
35–44	124 (87%)	2 (1%)	14 (10%)	0 (0%)	1 (0.5%)
> 45	36 (72%)	6 (12%)	2 (4%)	4 (8%)	1 (2%)

Three patients were found to have atypical lobular hyperplasia. One patient was 49 years old and had already had total abdominal hysterectomy and bilateral salpingo-oophorectomy with one paternal uncle with a history of an unknown malignancy. This patient received risk reduction counseling and was instructed to perform breast self-examination every 2–3 months with an annual breast exam from their primary care physician. A second 51-year-old patient with atypical lobular hyperplasia on a single pathology slide and a longstanding history of smoking and family history of breast cancer opted for 5 years of tamoxifen therapy for risk reduction. The third patient with atypical lobular hyperplasia was 20 years old and was lost to follow-up. One patient aged 34 years with an incidental locus of atypical ductal hyperplasia was found to have no additional loci on further analysis of their specimen and was advised to carry out the same self and clinical examinations described above. A 51-year-old patient was found to have ductal carcinoma in situ and was recommended to have additional surgery to remove breast tissue and to start endocrine therapy but declined treatment. One patient was found to have Paget’s disease on their top surgical specimen at the nipple terminal ducts and had the affected free nipple graft removed. The free nipple graft did not contain additional foci of cancer on pathologic analysis.

Ordered logistic regression analysis found that the variables of gender, smoking, alcohol use, personal or family history of cancer, and BMI were not associated with breast cancer risk in our cohort. Age was associated with increased risk, with those undergoing surgery at 45 or older having over a 300% increased risk of having an incidental finding on pathology compared with those aged 26–34 years [odds ratio (OR) 3.11, *p* < 0.01, confidence interval (CI) 1.37–7.09). This is in keeping with older age being a known risk factor for development of breast lesions and for our current institutional standard of required baseline mammographic screening of any patient 40 years and older prior to GAM. Additionally, patients aged 25 or younger were 70% less likely to have an incidental finding on pathological analysis with no patients in the present cohort having an occult malignancy (OR 0.3, *p* < 0.01, CI 0.18–0.50). GAHT use was not associated with an increased risk of having an incidental finding on pathological analysis. In the presented population, patients taking GAHT for a year or less were significantly less likely to have a pathological finding compared with those who were not (OR 0.5, *p* = 0.05, CI 0.25–0.99). This may represent an initial finding of the protective significance of testosterone therapy, although this does not hold true for the other cohorts and will require larger cohort studies (see Table 4).

**Table 4:** Ordered logistic regression parameters of demographic, lifestyle, and clinical factors associated with pathological risk of malignancy in breast specimens

Factor	Odds ratio	*p* value	95% confidence interval
Age (ref. 26–34 years)			
<25	0.3	**< 0.01**	0.18–0.50
35–44 years	1.05	0.87	0.54–2.08
45 years or older	3.11	**< 0.01**	1.37–7.09
Gender (ref. female to male)			
Male	0.57	0.1	0.32–1.10
Female	N/A	0.99	N/A
Nonbinary	0.88	0.65	0.49–1.56
Other	1.44	0.15	0.80–4.31
Tobacco use (ref. nonsmoker)			
Current smoker	0.67	0.23	0.35–1.28
Former smoker	0.73	0.21	0.44–1.19
Not specified	N/A	0.99	N/A
Alcohol use (ref. no alcohol use)			
Social alcohol use (1–2 times per month)	0.88	0.69	0.47–1.64
1–7 drinks per week	1.18	0.48	0.74–1.88
>7 drinks per week	2.94	0.052	0.99–8.72
BMI at time of surgery (ref. 21–25 kg/m^2^)			
Not specified	0.44	0.29	0.10–2.02
15–20	1.07	0.88	0.44–2.62
26–30	0.95	0.85	0.54–1.65
31–35	0.83	0.56	0.45–1.55
36-40	1.42	0.16	0.85–2.84
41–45	0.3	0.053	0.84–1.01
46–50	0.77	0.73	0.17–3.48
>50	1.1	0.91	0.20–6.17
Personal history of cancer	N/A	0.99	N/A
Family history of cancer	1.1	0.52	0.74–1.68
Duration of testosterone use in years (ref. nonusers)			
1 year or less	0.5	**0.05**	0.25–0.99
1–2 years	0.97	0.92	0.51–1.83
2–3 years	0.87	0.73	0.41–1.87
3 years or more	1.13	0.71	0.59–2.13

Bold values are statistically significant (*p* ≤ 0.05)

## Discussion

To our knowledge, the present study represents the largest single-center series of pathological gender-affirming top surgery samples of TGD patients. Of 1654 specimens, 123 were found to have incidental pathology with 34 patients having a proliferative lesion, 4 patients having proliferative lesions with atypia, and 2 having malignancies. The absolute number of cancers among this relatively young and healthy population is low. Typical risks for breast cancer still apply in transgender patients with age being the single most significant risk factor for breast cancer regardless of gender identity.^12^ Other studies seeking to identify risks in this population also found that older age was the primary risk and that TGD patients develop breast cancer at similar rates to their cisgender counterparts.^6,9,13^

Screening guidelines for breast cancer in TGD patients are lacking, with the American College of Radiology having the only formal guidance for clinicians to reference. These guidelines are somewhat vague, largely rely on expert opinion with data from a single study, and leave final shared decision making in the hands of practitioners and patients.^9,10^ Previous studies and the present data suggest that GAHT may not increase a patient assigned female at birth’s baseline risk for breast malignancy and should not affect screening guidelines for breast cancer in this population.^6,9,14^ Our study of the largest TGD pathologic specimen cohort suggests a similar risk of breast cancer development in this population of individuals assigned female at birth compared with their cis-gender counterparts. When it comes to screening recommendations, our institution screens all those undergoing GAM for breast cancer development risk. If there are patients of concern, we offer a multidisciplinary approach where they are referred to a breast surgeon who evaluates the patient’s risk of breast cancer development with the BCRAT from the National Cancer Institute. Those at high risk of breast cancer development (score > 20%) may benefit from additional mammographic and MRI screening prior to GAM. At our institution, this level of multidisciplinary care has led to several high-risk patients undergoing risk reducing mastectomies instead of GAM and one patient undergoing concurrent risk reducing mastectomies and chest masculinization with the combined efforts of the plastic surgeon and the breast surgeon.

Given the possibility of detecting possible malignancy in gender-affirming mastectomy patients, some advocate for pathological examination for every breast specimen.^15,16^ Oftentimes, detection of low-grade aberrant breast lesions does not alter immediate management or guide screening decisions. The present analysis found that those 25 years or younger in our cohort were 70% less likely to have any incidental pathology findings, suggesting that increasing age should be the central consideration for pathological evaluation. Therefore, it is our recommendation that high-risk patients based on BCRAT scores or those patients older than 25 years of age should have pathological analysis done on their breast tissue. Further investigation will likely show that this age recommendation is conservative but is substantiated by our analysis and reduces resource allocations.

The costs associated with pathological breast examination have been calculated at US$233 per specimen in the literature, and it has been suggested that cis-gender patients 18 years of age and younger are unlikely to benefit from pathological analysis.^17^ In our cohort these suggestions would have decreased the number of pathological analyses by as many as 600 breasts (300 patients) at an estimated savings of roughly US$140,000. This is particularly pertinent in this patient population who often pay out of pocket for GAM.

All studies have inherent limitations. The present sample is comprised of relatively young patients with few comorbid conditions from a single institution which may limit the generalizability of our results; however, current estimates show that our population is closely aligned with national samples of patients undergoing this surgery.^18^ Additionally, this study is retrospective in nature and long-term follow-up for all patients was not possible. All included patients had relevant pathology data and GAHT history, ensuring the presented data’s reliability and value.

## Conclusion

Data-driven consensus recommendations for breast cancer screening and pathologic analysis among TGD patients who have received GAM surgery are lacking. The present study sought to compile and analyze data to contribute to the generation of specific guidelines to manage breast cancer screening and pathologic specimen management in this population. Our findings suggest that the overall incidence of cancer in TGD patients seeking top surgery is low. Thus, tailoring screening to patients 40 and older or patients with higher risk of breast cancer development and pathological tissue analyses to patients above the age of 25 in a multidisciplinary management model may represent early steps towards conscientious resource allocation and more evidence-based patient management.
